# Measuring birth weight and umbilical cord diameter at birth to predict subsequent performance in swine

**DOI:** 10.1093/tas/txaa214

**Published:** 2020-11-19

**Authors:** Amanda L Fordyce, Elizabeth A Hines, Erika M Edwards, Suppasit Plaengkaeo, Kenneth J Stalder, Jessie D Colpoys, Jennifer M Bundy, Anna K Johnson, Howard D Tyler

**Affiliations:** 1 Department of Animal Science, Iowa State University, Ames, IA; 2 Department of Agricultural Science, Truman State University, Kirksville, MO; 3 Department of Animal Science, Pennsylvania State University, University Park, PA

**Keywords:** birth weight, market weight, piglet, pre-weaning mortality, umbilical cord, umbilical hernia

## Abstract

In the swine industry, pre-weaning mortality, umbilical hernia incidence and pig market weight are a few contributing factors affecting profitability and welfare on farm. Therefore, the ability to reliably predict any of these outcomes is valuable to swine operations. Mortality during the pre-weaning phase, umbilical hernia incidence and poor-quality finisher pigs can represent a multi-million dollar loss and increase in welfare concerns to the producer. Consequently, the objective of this study was to evaluate whether birth weight (BW), umbilical cord diameter at birth (UCD), and the calculated umbilical diameter at birth to birth weight ratio (UCD:BW), are potential indicators of both placental efficiency and relative defect size in the abdominal musculature as well as reliable predictors of pre-weaning mortality, umbilical hernia incidence, and pig body weight at 150 d of age in a commercial facility. Mixed sex commercial piglets were followed through production. Four hundred sixty-five piglets were weighed within 1 h of birth, and the UCD was determined using digital calipers, these animals were followed through weaning. Three hundred eighty-five pigs of the 465 were followed through the post-wean phase in the nursery facility and checked for umbilical hernia incidence. Finally, of the 385 pigs, 177 pigs were assessed for umbilical hernia incidence and weighed a final time at the grower-finisher facility. All data were analyzed using PROC Logistic and PROC GLM procedures. The variables of UCD:BW and BW were significantly associated with the probability of increased pre-weaning mortality (*P* < 0.001). For example, piglets with a low UCD:BW, but an increased BW had the greatest survival rate. Umbilical diameter (UCD) was not significantly associated with pre-weaning mortality. Post-weaning mortality was not significantly affected by UCD:BW, BW, or UCD variables. Umbilical hernia incidence was not significantly affected by UCD:BW at the nursery phase or growing-finishing phase. Pig body weight at 150 d of age was significantly affected by UCD:BW, BW, and UCD variables *(P* < 0.001). For example, piglets that had a larger UCD weighed more at 150 d of age. In conclusion, measuring the calculated UCD:BW has the potential to be a novel tool for future research looking into the impacts of umbilical measurements as it relates to placental function, fetal development, piglet survivability and impacts on future performance of the animal.

## INTRODUCTION

There are many factors that are considered important for future productivity and profitability of a commercial swine operation, namely keeping pigs alive and marketing a high percentage of top market pigs (Bilbrey, 2012). The ability to reliably predict or explain mortality probability and efficiency of gain are therefore valuable to swine operations. There is a vast body of literature surrounding the topic of pre-weaning mortality. Panzardi et al. (2013) documented that cyanotic skin, delayed time to stand after birth (>5 min), broken umbilical cords at birth, high birth order (>9), low birth weight (<1.3 kg), and low 24 h rectal temperature (<38.1 °C) are some common indicators of reduced viability and increased likelihood that individual piglets will die during the first week of life. Milligan et al. (2002) reported that the difference in survival rates between low and higher neonatal weight piglets was greatest in litters from older sows and in litters of 12 or more piglets. Neonatal weight variation within litter was a significant pre-weaning mortality predictor, independent of factors such as mean neonatal weight, litter size, and parity of the sow (Pettigrew et al., 1986; Milligan et al., 2002). However, even though within-litter variation was associated with increased risk of pre-weaning mortality, Fix et al. (2010a) reported that individual birth weight was a better predictor.

Umbilical hernias ultimately result in a multi-million dollar loss to the swine industry due to reduced growth potential, low-value pigs at weaning and harvest and higher grow to finish animal culling rates (Ding et al., 2009; Anderson and Mulon, 2019). Umbilical hernias are defined as a discontinuity of the abdominal wall at the umbilicus region with protrusion of abdominal content into the skin and surrounding connective creating a hernia sac (Anderson and Mulon, 2019). Umbilical hernia prevalence has been reported as low as 0.4% and up to 1.5% in commercial herds (Searcy-Bernal et al., 1994; Straw et al., 2008; Ding et al., 2009; Yun et al., 2017). The etiology and genetic influence on the incidence rate of umbilical hernias is difficult to precisely define and is theorized to be related to a genetic predisposition to weak or compromised abdominal musculature or abnormal collagen formation in the umbilical region (Searcy-Bernal et al., 1994; Straw et al., 2008; Ding et al., 2009). Moreover, the addition of proper umbilical cord sanitation efforts and maintaining pen hygiene may offer additional effective measures to decrease the incidence of umbilical hernias by decreasing navel infection and abscesses (Straw et al., 2008; Anderson and Mulon, 2019). Therefore, the current proven theories remain centered around an interaction of genetics, environment, and management related factors.

The challenge for researchers is delineating easily measured factors early in the piglet's life that reliably predict profitability and welfare decisions for the producer. The producer's top priority is the pig's ability to reach full value, which is dependent on mortality, adequate growth to reach weight requirements and pig quality at time of harvest (Fix et al., 2010a). Therefore, the objective of this study was to evaluate newborn piglet birth weight (BW), umbilical diameter at birth (UCD), and the calculated umbilical diameter to birth weight ratio (UCD:BW) as predictors of pre-weaning mortality, umbilical hernia incidence rate, and body weight at 150 d of age in a commercial facility.

## MATERIALS AND METHODS

### Animals and Data Collection

The Iowa State University Animal Care and Use Committee (IACUC) approved this study. Four hundred sixty-five piglets (DNA 600 Genetics Duroc boar x PIC 1050 Camborough sow) were initially enrolled in this study at farrowing time on a commercial farm in January 2015. Steel farrowing stalls were approximately 2.1 × 1.5 m, with a heat lamp over one rubber mat (approximately 0.3 × 0.5 m) on one side of the stall used to provide supplemental heat to the piglets after birth. Piglets were born immediately behind the sow and were able to move freely within the farrowing stall after birth. After birth, piglets were determined initially as either viable or nonviable. The definitions of nonviable piglets followed the farm standard operating procedures and included piglets deemed very weak, that died shortly after birth, stillborn or had cyanotic skin. All nonviable piglets were excluded from this study.

Because the commercial facility did not individually identify piglets, viable piglets had a button ear tag (Allflex USA Inc., DFW Airport, TX) placed in the ear to individually identify each piglet for the purposes of this study. The UCD was determined by measuring the diameter of the umbilical cord approximately 10–15 mm below the umbilical stump using digital calipers (Mitutoyo – 500-197-30 – Absolute Digital Caliper, Aurora, IL), and individual BW was recorded prior to nursing by placing the piglet on the scale for approximately 1 min (Way Pig Portable Litter Scale, Mechanical, Raytec Manufacturing, Ephrata, PA). Sex of each piglet was also recorded.

Piglets were weaned at an average of 17–18 d and were transported to a nursery site where they were housed in groups and fed an appropriate ration (NRC 2012 and lysine levels within normal range) and had *ad libitum* water access. At 12–14 wk of age, pigs were transported to a grow-finish site where they were housed in large groups, fed an appropriate ration (nutrient levels met or exceeded NRC 2012 recommendations) and were provided *ad libitum* water access. Both nursery and grow-finish barns had slatted concrete floors, deep pits, curtain sides, and housed approximately 2,500 pigs. Mortality rates were reported by the site manager at two time points: 1) pre-weaning (piglets 10 d through 17–18 d age) and 2) final (post-weaning 146–152 d age).

Of the 465 piglets originally enrolled in the study at birth, 177 pigs completed the study at 150 d of age. Pigs exited the study prematurely due to pre-weaning mortality (13.5%), post-weaning mortality (3.9%), culling, and euthanasia (2.5%), or loss of button-ear tag identification (42.9%). Despite these losses, EPV calculations were used to statistically verify that the data is not biased by any of the loss reasons stated and significant sources of variation contributing to dependent trait variability were able to be viably determined with the pigs that remained on trial.

Visual umbilical hernia checks were done at two time points in the production cycle; 10 wk of age and at 20 wk of age. Pig body weight measurements were performed on the remaining 177 pigs at 150 d of age by two trained researchers at the finishing site. Umbilical hernia sacs were noted by visual observation by kneeling next to the individual pig before the animal entered the floor scale (Digi-Star SW600 (scale head), Digi-Star, LLC, Fort Atkinson, WI). This procedure is an approved umbilical hernia diagnosis method and may be followed with palpation and ultrasonography if needed (Pearson, 2016). The smallest recorded hernia in this study was a baseball size (this size was determined as the minimum size one could visually deem a hernia without the use of palpation or ultrasound methods). Overall, hernia sizes ranged from baseball, small football, to a soccer ball in size. A bulge near the umbilicus region smaller than a baseball was deemed “non-herniated” by the researchers for the purposes of this study. Pigs with a baseball size or smaller hernia were still able to be processed as full value. Anything larger was sent to a cull market. Overall, 12 of the 177 pigs assessed had an identifiable hernia at 146–152 d old (6.7% incidence rate).

### Statistical Analysis

All analyses were performed with SAS University Edition (SAS/STAT, SAS Institute Inc, NC, USA) using PROC LOGISTIC and PROC GLM procedures. Descriptive analysis was obtained with MEANS procedure. Models included the fixed effects of sex. A binary logistic model was used to model the probability of pre-weaning mortality and umbilical hernia incidence, where UCD:BW, BW, and UCD were continuous variables and pre-weaning mortality and umbilical hernia incidence were considered as binary variables. A general linear model was used to model the pig body weight at 150 d of age as a dependent variable and UCD:BW, BW, and UCD as independent variables. Additionally, a general linear model was used to analyze the covariance of BW and UCD and to group UCD values into quartiles to assess trends of BW means within a quartile group.

### Data Description

Animals analyzed at each major time point in production through birth to finish were as follows: pre-weaning mortality, and prenatal values (UCD:BW, BW, and UCD), *n* = 465 animals; post-weaning mortality reported by site manager, *n* = 403 animals; umbilical hernia incidence at 10 wk old, *n* = 310 animals; umbilical hernia incidence at 20 wk old, and body weight at 150 d of age, *n* = 177.

## RESULTS

Piglet BW was measured prior to colostrum intake; therefore, BW values used in calculating UCD:BW were not impacted by colostrum volume ingested by individual piglets. Birth weight for viable piglets ranged from 0.46 to 2.66 kg with a mean BW of 1.15 ± 0.33 kg. Umbilical cord diameter ranged from 3.76 to 11.58 mm with a mean UCD of 6.62 ± 1.19 mm. The calculated UCD:BW ranged from 2.17 to 11.96 mm/kg with a mean ratio of 6.09 ± 1.56 mm/kg. Descriptive analysis results are presented in Table 1.

**Table 1.** Descriptive statistics of piglets evaluated at birth and body weight at 150 d of age on a commercial farm

Item	*N*	Mean	SD	Min	Max
Birth weight, kg	465	1.15	0.33	0.46	2.66
Umbilical cord diameter, mm	465	6.62	1.19	3.76	11.58
Umbilical diameter to birth weight ratio, mm/kg	465	6.09	1.56	2.17	11.96
Body weight at 150 d of age, kg	177	89.98	13.91	51.26	126.55

**Figure 1.**
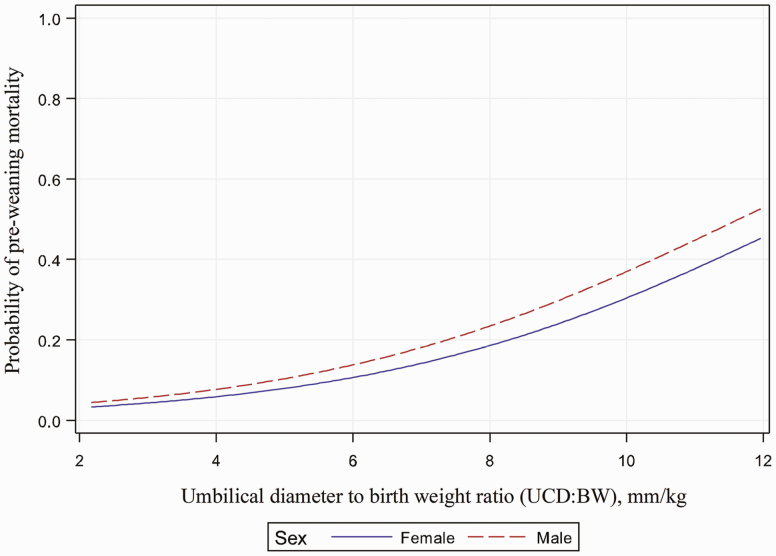
Association between umbilical diameter to birth weight ratio and probability of pre-weaning mortality. Graph of the logistic regression curve showing UCD:BW predicting the probability of pre-weaning mortality. For every one-unit change in UCD:BW ratio, the log odds of pre-weaning mortality increases by 0.325. Piglets with a high UCD:BW ratio have a greater probability of pre-weaning mortality when compared with piglets with a low UCD:BW ratio (*P* < 0.001).

**Figure 2.**
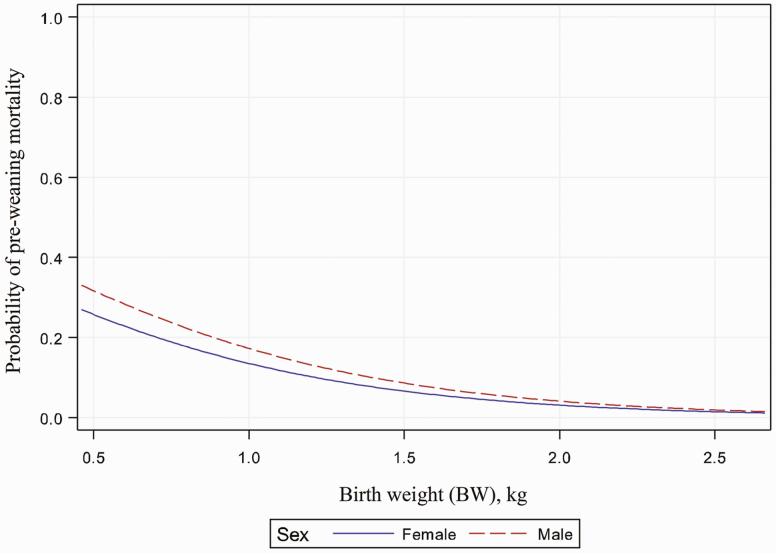
Association between piglet birth weight and probability of pre-weaning mortality. Graph of the logistic regression curve showing probability BW predicting pre-weaning morality. For every one-unit change in BW, the log odds of pre-weaning mortality decreases by −1.597. Piglets with a high BW have a lower probability of pre-weaning mortality when compared with piglets with a low BW (*P* < 0.001). Female piglets with a BW >1.5 kg have a probability of pre-weaning mortality of <7%.

**Figure 3.**
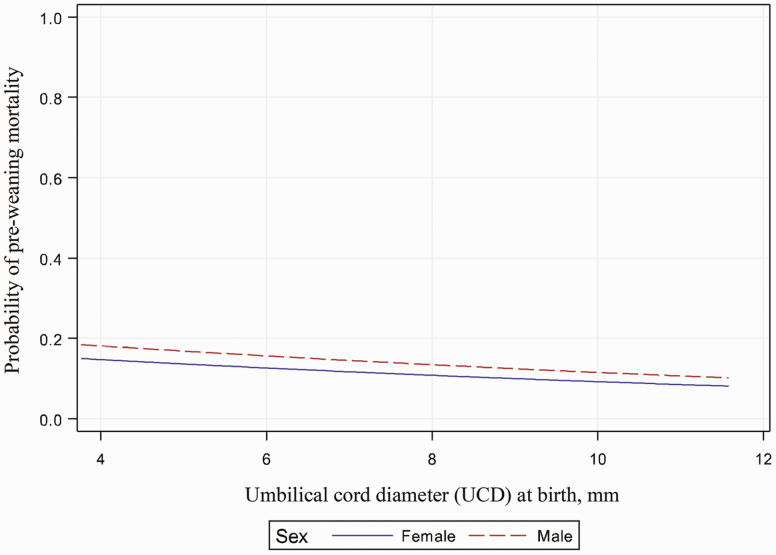
Association between umbilical cord diameter at birth and probability of pre-weaning mortality. Graph of the logistic regression curve showing probability of UCD predicting pre-weaning morality. Pre-weaning mortality was not significantly affected by UCD or Sex (*P* = 0.46 and *P* = 0.38, respectively).

The logistic analysis revealed that pre-weaning mortality was significantly affected by UCD:BW and BW (*P* < 0.001; Figures 1 and 2), while pre-weaning mortality was not significantly affected by UCD (Figure 3). The BW, UCD, and UCD:BW were divided into 4 groups (lower quartile = Q1, lower quartile to median = Q2, median to upper quartile = Q3, upper quartile = Q4). When UCD:BW was classified into quartiles, piglets from UCD:BW Q4 had higher odds (*P* < 0.01) of mortality when compared to piglets from UCD:BW Q1, while piglets from UCD:BW Q2 and Q3 had no difference when compared with piglets from UCD:BW Q1 (Table 2). For example, female piglets with a UCD:BW > 10 mm/kg had a probability of pre-weaning mortality (>30%) when compared to female piglets with a UCD:BW ≤ 0.9 mm/kg.

**Table 2.** Results of logistic regression analysis for BW, UCD and UCD:BW, and probability of pre-weaning mortality

Variables	Odds ratio	95% confidence limits	*P*-value
Birth weight (BW)*, kg			
Q1 vs. Q2	0.591	0.302–1.155	0.1241
Q1 vs. Q3	0.248	0.107–0.572	0.0011
Q1 vs. Q4	0.364	0.171–0.774	0.0087
Umbilical cord diameter (UCD)^†^, mm			
Q1 vs. Q2	1.408	0.694–2.857	0.3429
Q1 vs. Q3	0.820	0.376–1.792	0.6196
Q1 vs. Q4	0.820	0.376–1.792	0.6196
Umbilical diameter to birth weight ratio (UCD:BW)^‡^, mm/kg			
Q1 vs. Q2	1.662	0.690–4.007	0.2576
Q1 vs. Q3	1.384	0.560–3.421	0.4814
Q1 vs. Q4	3.898	1.748–8.693	0.0009

*Birth weight (BW) was divided into 4 quartile groups: Q1 = BW ≤ 0.9 kg, Q2 = BW 1.0 to 1.13 kg, Q3 = BW 1.14 to 1.34 kg, and Q4 = BW ≥ 1.35 kg

^†^Umbilical cord diameter (UCD) was divided into 4 quartile groups: Q1 = UCD ≤ 0.9 mm, Q2 = UCD 1.0 to 1.13 mm, Q3 = UCD 1.14 to 1.34 mm, and Q4 = UCD ≥ 1.35 mm

^‡^Umbilical diameter to birth weight ratio (UCD:BW) was divided into 4 quartile groups: Q1 = UCD:BW ≤ 0.9 mm/kg, Q2 = UCD:BW 1.0 to 1.13 mm/kg, Q3 = UCD:BW 1.14 to 1.34 mm/kg, and Q4 = UCD:BW ≥ 1.35 mm/kg

When BW was classified into quartiles, piglets from BW Q3 and BW Q4 had lower odds (*P* < 0.01) of mortality when compared to piglets from BW Q1, while piglets from BW Q2 had no difference when compared with piglets from BW Q1 (Table 2). For example, the odds of mortality are 40% lower for piglets from BW Q2 (1.14 to 1.34 kg) when compared to BW Q1 (<0.90 kg). On the other hand, when UCD was classified into quartiles, piglets from UCD Q2, Q3, and Q4 had no difference when compared with piglets from BW Q1 (Table 2).

**Figure 4.**
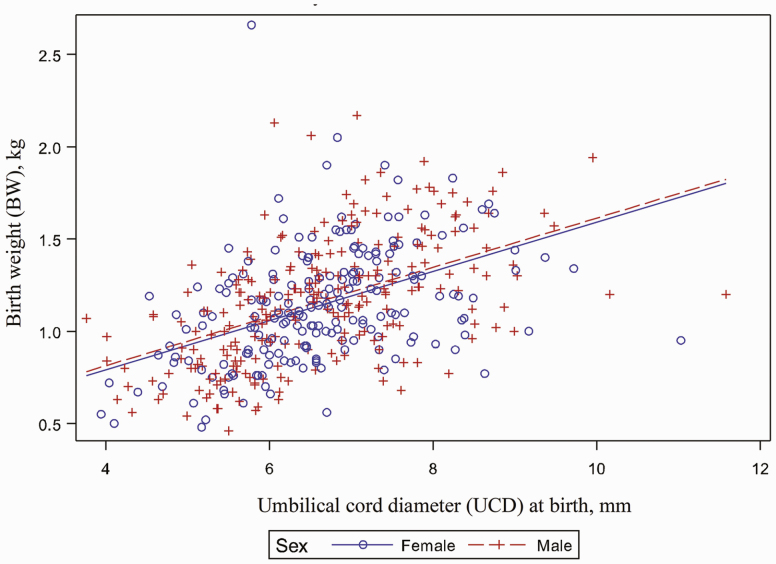
Relationship between umbilical cord diameter at birth and birth weight. Umbilical diameter has a statistically significant effect on birth weight of piglets (*P* < 0.0001). As umbilical diameter increases, birth weight also increases. Larger birth weight piglets have a larger diameter umbilical cord at birth.

**Figure 5.**
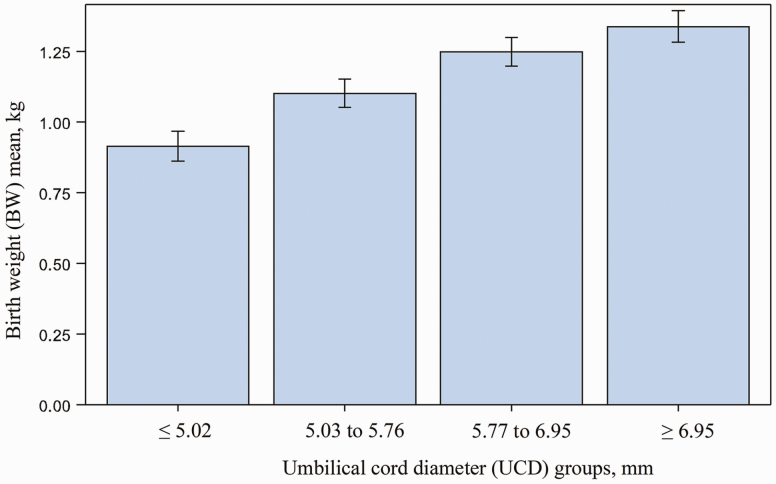
Birth weight means within an umbilical diameter quartile group. When UCD values were organized into quartile groups, BW means within each group were statistically different (*P* < 0.0001 for all groups comparisons except group 3 and 4, *P* = 0.04). Piglets had a greater mean BW (1.33 kg) in UCD group 4 when compared to piglets who had the lowest BW mean (0.92 kg) in UCD group 1.

Birth weight was significantly affected by UCD (*P* < 0.001; Figure 4) When UCD values were organized into quartile groups, BW means within each quartile group were statistically different (*P* < 0.001) for all group comparisons except Q3 and Q4 (*P* < 0.05; Figure 5). For example, piglets from UCD Q4 had significantly greater mean BW (1.33 kg) when compared to piglets from UCD Q1 which had the lowest mean BW (0.92 kg).

Umbilical hernia incidence of pigs at either time point (10 and 20 wk of age) was not significantly affected by UCD:BW, BW or UCD. Additionally, post-weaning mortality (reported by site manager when pigs ranged between at 12–14 wk old) was not significantly affected by UCD:BW, BW or UD.

**Figure 6.**
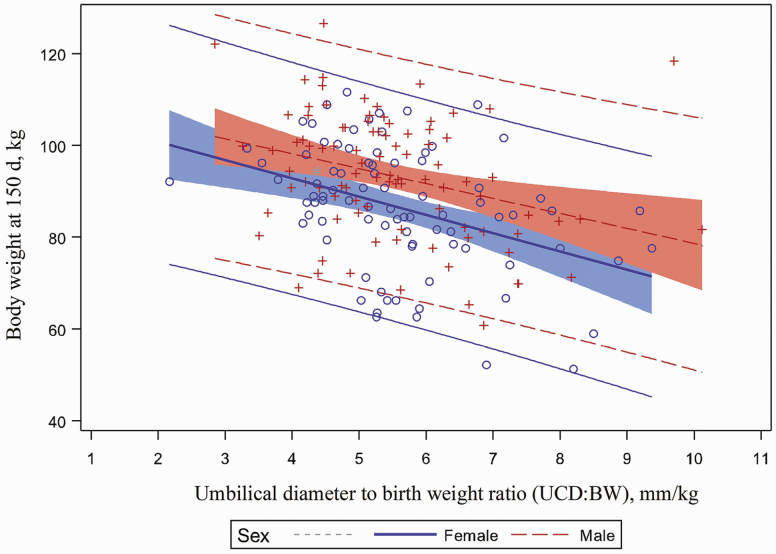
Relationship between body weight at 150 d and umbilical diameter to birth weight ratio. Pig body weight at 150 d of age was affected by umbilical diameter to birth weight ratio *(P* < 0.0001). Piglets with the highest ratio of umbilical cord diameter to birth weight weighed less at 150 d of age compared to piglets with a lower ratio of umbilical cord diameter to birth weight.

**Figure 7.**
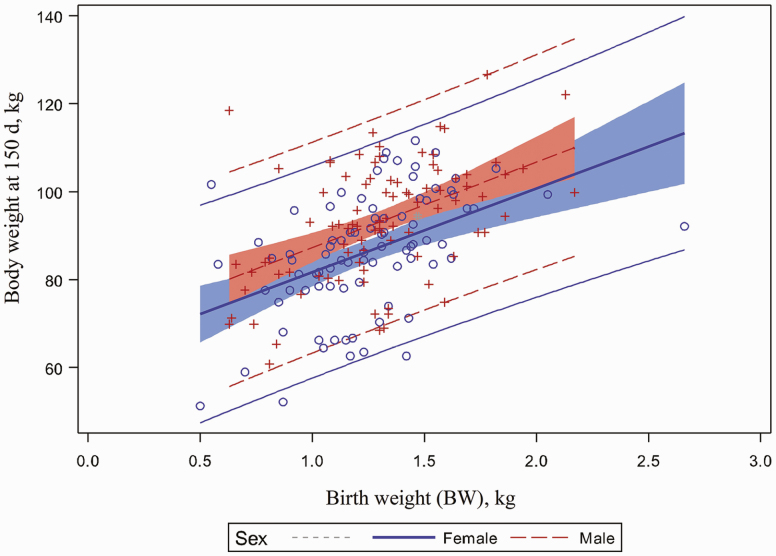
Relationship between body weight at 150 d and birth weight. Pig body weight at 150 d of age was significantly affected by birth weight of piglets (*P* < 0.0001).

**Figure 8.**
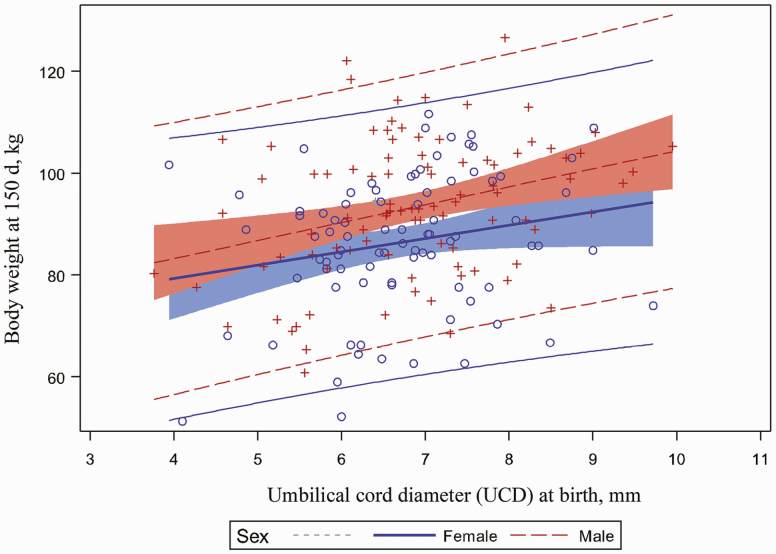
Relationship between body weight at 150 d and umbilical cord diameter at birth. Pig body weight was significantly affected by UCD measured at birth (*P* = 0.0003).

Pig body weight at 150 d of age was significantly affected by UCD:BW (*P* < 0.001; Figure 6). Piglets with a higher UCD:BW weighed less at 150 d of age. A favorable relationship between pig body weight at 150 d of age, BW and UCD was observed in this study (*P* < 0.001). Piglets with the highest BW and largest UCD weighed more at 150 d of age (*P* < 0.001, Figure 7 and *P* < 0.001, Figure 8, respectively). In addition, barrows had a greater market weight when compared to gilts (*P* < 0.001). Pig body weight at 150 d of age was not affected by umbilical hernia incidence at 10 or 20 wk of age.

## DISCUSSION

Pre-weaning mortality is influenced by many factors: genetics, farrowing duration, birth order, birth weight, sex, litter size, environment after birth (thermal environment and hygiene status), nutritional status, disease prevelance, and sow and piglet behavior (Lay et al., 2002; Panzardi et al., 2013). Piglets born with low birth weights are considered physiologically compromised due to decreased energy stores and susceptibility to hypothermia, and disadvantage to competing at the udder of the sow (Lay et al., 2002). In agreement with Fix et al. (2010a), our study showed increased birth weight was associated with a reduced chance of mortality prior to weaning. Increased pre-weaning mortality due to low birth weight could be caused by a variety of prenatal developmental and postnatal environmental factors (Fix et al., 2010a). Many prenatal factors have been documented to affect pospartum death such as; uterine capacity, placental vascularity and function, umbilical cord morphology, and fetal development (Foxcroft et al., 2006; Rootwelt et al., 2013).

Swine have a diffuse placental type and each fetus has an individual placenta with avascularized tips preventing sharing or partitioning of blood, nutrients, and hormones. This means each piglet and placenta develops independently and placental efficiency can vary within each litter. Randall et al. (1989) reported that umbilical cord length at term ranged from 17 to 50 cm and was positively correlated with piglet body weight. Umbilical cord length is observed to be highly variable within litters and littermates, but the cause for this variation remains unclear (Randall et al., 1989). Absolute placental blood flow increases with fetal size (Dawes, 1968), and this increased blood flow and pressure could provide tension within the umbilical vessels during late gestation stimulating the cord to lengthen (Randall et al., 1989). Umbilical cord length, strength, and elasticity varies between piglets and between litters; however, the factors affecting diameter variation have yet to be documented in animals. In human infants, umbilical diameter varies due to the amount of Wharton's jelly matrix within the cord and the umbilical artery thickness (Proctor et al., 2012). Additionally, Proctor et al. (2012) documented that thin UCD in infants may contribute to the spectrum of placental insufficiency leading to fetal growth restriction. In the current study, piglet umbilical diameter was measured at birth rather than umbilical length, unlike any previous study in the scientific literature. In agreement with previous literature discussed by Randall et al. (1989) and Proctor et al. (2012) our data showed that as UCD increases, BW also increased, and there was a statistically significant co-variance between the UCD and BW variables suggesting a relationship between the two variables. Physiologically, this makes sense, the umbilical cord serves as a vascular conduit for substrates to move between the placenta and the fetus (Proctor et al., 2012).

Umbilical diameter has been shown to be related to both placental weight and birth weight in human infants (Di Naro et al., 2001; Proctor et al., 2012) and therefore was used to formulate the umbilical diameter to birth weight ratio in the present study. Umbilical diameter has also been hypothesized to be related to placental efficiency in humans (Proctor et al., 2012). Calculating the ratio of umbilical diameter to birth weight may provide an indirect placental efficiency estimate and serve as a substitute for placental weight to piglet birth weight data. This efficiency estimate method has been previously documented and validated in infants (Proctor et al., 2012). The traditional method to track individual piglets is accomplished by double ligating the umbilical cord with a color code at farrowing, cutting between the ligations and waiting for the placentae to be expelled (Rootwelt et al., 2013). However, once validated, the UCD:BW may eliminate the need to identify and match individual placentas and piglets, which is labor intensive due to random birthing order and varying placenta detachment times.

Placental efficiency has been defined as the ratio of birth weight to placental weight (Knight et al., 1977). Placental efficiency is an important factor involved in fetal development and subsequent postnatal development. Knight et al. (1977) reported that placental weight and fetal weight are strongly associated, and placental weight alone was as accurate of a predictor of fetal weight as a summation of documented individual traits such as; placental surface area, placental length or areolae surface area. Rootwelt et al. (2012) found that the piglet is a product of the placenta, where piglet BW was positively associated with placental area and placental weight. Furthermore, it has been concluded that birth weight was the only accurate predictor of pre-weaning mortality (van Rens et al., 2005). However, our data has illustrated that increases in the UCD:BW resulted in lower pre-weaning survival rates and decreased weight around the time of the finishing phase, potentially reflecting altered vasculature and compromised function of the pig postnatally. This hypotheses that the effect of birthweight on future body weight is likely attributable to both prenatal and postnatal factors associated with differences in birthweight are confirmed by several studies (Foxcroft et al., 2006; Fix et al., 2010a, 2010b; Yun et al., 2017).

An additional major topic in swine production is the subject of intrauterine growth restriction (IUGR) and resulting pig production (Foxcroft et al., 2006; Yun et al., 2017). Intrauterine growth restriction is defined as reduced growth and development of the mammalian embryo/fetus or its organs during gestation (Wu et al., 2006). Uterine crowding early in the post implantation period results in detrimental effects on placental development (Foxcroft et al., 2006). Due to this early crowding in utero and placental development adaption, this limits nutrient availability to the embryo during a critical period of muscle development. This compromised development results in IUGR and reduced numbers of muscle fibers at d 90 and at birth, in all surviving littermates (Foxcroft et al., 2006). Together with the earlier literature, our results (when analyzing the UCD:BW and UCD variable effects on body weight at 150 d of age) supports the suggestion that prenatal imprinting due to placental function, efficiency, and potential IUGR causes decreased muscle fiber development causing smaller birth weight piglets. In addition to having decreased muscle fiber development, runt piglets may have a birth weight one-half or even one-third as much as larger littermates, which means organs involved in nutrient utilization are disproportionately smaller and negatively affected compared to larger littermates (Wang et al., 2008; Yun et al., 2017). This may help to explain why birth values such as the ones measured in this study (BW, UCD, UCD:BW) had statistically significant impacts on 150 d weight. In agreement with previous literature discussed, data from our study showed that surviving piglets that had a low birth weight and small umbilical diameter had a lower chance of survival during the pre-weaning period, and also weighed less at 150 d of age. Additionally, piglets with a high calculated UCD:BW had a greater chance of pre-weaning mortality and weighed less at 150 d of age.

The etiology of swine umbilical hernias has long been debated by veterinarians, researchers, and producers. The most common theories involve environmental factors and the possible interaction of genetics and environmental factors (Ding et al., 2009). The genetic control for umbilical hernia incidence appears to be much less than that seen with inguinal or scrotal hernias (Ding et al., 2009). It is notable that a 6.7% umbilical hernia incidence rate was recorded for pigs enrolled in this study which is considered high, compared to previously reported incidences ranging from 0.4 to 1.5% in commercial herds (Searcy-Bernal et al., 1994; Straw et al., 2008; Ding et al., 2009; Yun et al., 2017). Genetic backgrounds of the pigs included in this study were Durocs, which Ding et al. (2009) documented to have an increase in hernia incidence compared to other breeds. Their research characterized susceptibility loci for occurrence of scrotal/inguinal and umbilical hernias by a genome-wide scan in a White Duroc × Erhualian F2 resource population. Rutten-Ramos et al. (2006) used progeny testing of 25 single-sire litters that could identify potentially heritable defects, which occurred double of what was noted in the normal population, showing that umbilical hernias may be associated with genetic lineage. However, compared to other types of hernias, there appears to be a much higher interaction between genetics and environmental factors for umbilical hernias. The environmental factors are presumed to be; umbilical infection rate, navel sucking, and umbilical stretching at farrowing (Ding et al., 2009). Researchers have reported that an unhygienic farrowing environment may lead to a bacterial infection of the umbilical stump, which may potentially lead to a failure in closing or healing of the umbilical cord (Searcy-Bernal et al., 1994; Anderson and Mulon, 2019).

Straw et al. (2008) found that among pigs with umbilical hernias, neither defect prevalence or mortality rate differed between gilts and barrows. Furthermore, in contrast to this study’s findings, pigs with hernias (independent of size) at 80 d of age had a slower growth rate compared to non-hernia pigs. It was also documented that pigs with the largest umbilical hernias (approximately melon size) had similar ADG compared to pigs with medium (approximately baseball) or small (approximately golf ball) umbilical hernias (Straw et al., 2008). Although this study did not document ADG through production, it is notable that umbilical hernia incidence at 10 wk of age and 20 wk of age had no effect on body weight at 150 d of age.

Despite the growing body of research, there is still only limited data on umbilical hernia formation. In humans, the “Unified Hernia Theory” was developed, which progressed from the concept of increased intra-abdominal pressure overwhelming a weak abdominal wall into the complex intertwining of several basic sciences to explain one final common pathway, the collagen matrix, and syndromes of collagen disease. However, similar to swine, the story is not complete or refined (Bendavid, 2004). Interestingly, data from our study showed no statistically significant effect of UCD or UCD:BW measurements on incidence of umbilical herniation at 10 or 20 wk old. This disproves our original hypothesis that umbilical hernia incidence may be exasperated by a larger UCD on a smaller BW piglet. We hypothesized the calculated UCD:BW of the piglet may provide a novel, indirect measure related to the potential abdominal defect size. Increased umbilical vessel diameter at birth compared to the relative size of the individual piglet may contribute to an increased chance of developing umbilical hernias during the growing phase due to prolonged closure of the umbilical ring, increasing the opportunity for the intestines to protrude through the abdominal wall. However, data from this study did not confirm significance of umbilcal hernia predisoposition due to prenatal measures of UCD, BW or the calculated ratio between the two variables (UCD:BW). Future studies involving a greater sample population may have more opportunity to explore this hypothesis.

In summary, the findings from the present study indicate that the UCD:BW variable developed by taking two independent but physiologically linked variables to create a novel variable that has the potential to be a production tool for predicting future performance variables in pigs may serve as a point of interest to researchers on further explaining pre-weaning survival outcomes and variations in 150 d weight. The current study documents that the UCD:BW variable and BW are significant predictors of pre-weaning mortality and pig body weight at 150 d of age. Piglets that exhibited an increased UCD:BW had decreased survival during the pre-weaning phase and decreased weight at 150 d of age. Therefore, it is theorized that the UCD:BW can serve as an additional indicator of overall pig performance and can serve as the beginnings of further studies identifying the mechanisms involved in these effects such as; prenatal imprinting, placental function, pre-weaning mortality, growth outcomes, and market weight.
